# Evolution of HIV prevalence and behavioral factors among MSM in Togo between 2011 and 2015

**DOI:** 10.11604/pamj.2017.28.191.11285

**Published:** 2017-11-01

**Authors:** Julienne Noude Teclessou, Séfako Abla Akakpo, Koumavi Didier Ekouevi, Georges Koumagnanou, Assétina Singo-Tokofai, Palokinam Vincent Pitche

**Affiliations:** 1 Service of Dermato-Venerology, CHU Sylvanus Olympio, Unversity of Lome, Togo; 2 Public Health Department, University of Lomé, Togo; 3 Population Service International, Washington, US; 4 National AIDS Program, Ministry of Health, Saudi Arabia; 5 National AIDS Council of Togo, Togo

**Keywords:** HIV, behavioral MSM, Togo

## Abstract

**Introduction:**

The aim of this study was to assess sexual behavior and measure HIV prevalence among MSM in 2015, in Togo.

**Methods:**

We conducted a cross-sectional study from February to March 2015 in nine major cities of Togo. The respondent-driven sampling method was used to recruit MSM. Behavioral data were collected by interviewer-administered questionnaires. The blood tests were then carried out among MSM to assess their HIV status. Data were inputted into an Epidata database and exported to STATA^®^ 9.0 for analysis. Qualitative variables were compared using the chi-2.

**Results:**

A total of 496 MSM were involved in this study, with 43.35% in the capital, Lome. Over the past 12 months, 88.9% of MSM had had sexual intercourse with men and 24.1% of them had had sex with women. The last sexual intercourse was with a casual partner among 52.9% of MSM. During the last 30 days preceding the survey, 68.5% of MSM had regularly used a condom during active anal intercourse and 71.9% had used it during passive anal intercourse. The national prevalence rate of HIV among MSM was 13.0%. The factors associated with HIV infection were age of MSM OR = 5.30 [1.85-15.1], HIV testing history OR = 2.63 [1.18-5.87] and the city of residence of MSM OR = 5.56 [2.90-10.64].

**Conclusion:**

This study confirms that HIV prevalence among MSM is five times higher than in the general population (13% vs 2.5%). Thus, the need to rethink HIV sensitization and prevention strategies targeting hidden and stigmatized populations such as MSM.

## Introduction

Globally, HIV infection rates among men having sexual intercourse with other men (MSM) are far higher than among the general population [1]. Sero-epidemiological surveys conducted in Western European countries reveal an increase of more than 50% of new HIV positive cases among MSM between 2001 and 2004 [2]. In sub-Saharan Africa, reliable data are scarce and difficult to obtain because of stigma and socio-cultural taboos linked to homosexuality [3]. However, some studies conducted in Africa in this regard reveal an HIV prevalence rate 3 to 4 times higher than that noticed among the general population [4]. Thus, in South Africa, in 2008, HIV prevalence was estimated at 49.5% among 204 MSM screened in Johannesburg and 27.5% among 81 MSM screened in Durban [5]. In Senegal, two surveys were conducted among MSM and revealed an HIV prevalence rate of 17.5% in 2004 and 13% in 2007, while it was only 1% in the general population at the same period [6, 7]. Finally, in Nigeria, a national survey of 879 MSM revealed a 1.1% prevalence rate in Cross River State, 9.3% in Kano and 17.4% in Lagos [8]. Like Togo, many countries do not yet have a national surveillance system for most-at-risk populations. The only surveillance data available are sentinel sero-surveillance results obtained from pregnant women aged 15 to 49 years during antenatal consultations. Latest estimates revealed a prevalence rate of 2.5% in 2013 [9], indicating a significant decrease both by age groups as well as by geographic zones. However, the spread of the epidemic among highly exposed populations such as MSM, sex workers (SWS) and injectable drug users is still unknown. In 2011, a prevalence study conducted in six major localities in Togo among 758 MSM recruited through the snowball method revealed an average HIV prevalence rate of 19.6% [10]. The aim of this study, four years after the previous one, is to assess sexual behavior and measure HIV prevalence among MSM in Togo, in 2015, and to assess the impact of interventions implemented among this population since 4 years in the country.

## Methods

**Type and period of study**: Tt was a transversal study with a descriptive and analytic orientation, conducted from February to March 2015, in Togo, among a sample population recruited using the Respondent Driven Sampling (RDS) method.

**Survey population, inclusion and exclusion criteria**: The survey population basically comprised of males who openly stated having had sexual intercourse with other men, aged 18 and above, having lived in Togo for more than 3 months, regardless of nationality. Sexual ambiguities or transgender persons were excluded from this study. people with sexual ambiguities that are difficult to identify as male or female sex and trans-sexual people with disorders of sexual identity and who identify themselves as the opposite sex to their bodies were excluded.

**Choice of sites**: The survey was conducted in nine major cities of Togo. Major cities are those with at least 50,000 inhabitants and all cities that share borders with one of Togo's neighbors (Benin, Ghana and Burkina-Faso), regardless of their population. There are nine major cities in Togo. The following cities were chosen for this survey, from north to south: Sinkance, Dapaong, Kara, Sokode, Atakpame, Kpalime, Aneho, Tsevie and Lome. These cities were identified during a mapping study as having meeting hotspots for MSM, with an estimate of the national population of MSM and a per city estimate of MSM [11].

**Recruitment of MSM**: The RDS method was used to recruit MSM. It is a sampling method based on referral by MSM peers, known for its efficiency in HIV behavioral and biological surveillance among most-at-risk groups that are difficult to reach using classical methods. To start with, 25 resource persons from the MSM community were chosen as first interviewees (“seeds”). The choice of these “seeds” was motivated by their representation of the MSM social sub-groups in the study cities that is: insertive (tops) and receptive (bottoms), versatile, bisexual and gay. After their first interviews, these “seeds” were assigned to each recruit three other MSM from their social network using a coupon with a unique code. Thus, those recruited by the "seeds" were the first wave of participants and who, in turn, recruited a maximum of three (03) interviewees. The procedure continued until the ideal sample population required for the study was attained.

**Calculation of the sample size**: The sample size was calculated on the basis of estimates of prevalence for a descriptive study using the following formula and taking into account the included parameters:

$$\text{N}=\frac{\text{Z}[\text{P}(1-\text{P})]}{{△}^{2}}$$

With an expected HIV prevalence rate p estimated at 20% among MSM, going by prevalence data of 2011; a precise estimate of Δ at 4% and a first category risk of alpha α of 5%. The minimum sample size should be equal to 384 MSM.

**Data collection**: Behavioral data was collected using individual questionnaires with closed-and semi-closed-ended questions. A standardized questionnaire with the following variables was given to each MSM. It included the following components: socio-demographic data: MSM age at the time of investigation, the nationality of the MSM that is his country of origin. The level of education is the education level reached by the MSM; the professional activity is the activity generating an income or the work performed by the MSM; the Married MSM are those that have been legally Married in an institution acknowledged by the administrative authority and MSM concubine are those living under the same roof but have not contracted the wedding link. MSM living with family are those who are living with biological parents or with their spouses: risky sexual behaviors. Risk behavior involved alcohol or drug consummation, the frequency of sexual relations with men or with women by the MSM, the type of sexual intercourse; means of HIV and STI prevention: It concerns the use of condoms during sexual intercourse; HIV screening history; knowledge of HIV status. We investigated the social life of MSM including membership of an association of HSH, the contraction of gay wedding. An MSM association is a club where MSM meets periodically to share elements of their social life. Two MSM are married when they are hold by a legal Wedding unit link. Sexual risk behaviors of MSM in particular taking alcohol, drugs, the age of sexual partner, type of sexual intercourse and sexual partners the twelve months preceding the survey were evaluated.

**Ethical and statutory aspects**: The ensuing protocol and amendments were submitted by bioethics committee of the Health Minister of Togo. Their approval has obtein before the implementation of the study. A written and verbal informed consent was obtained prior to the survey that started with an interpersonal interview with each respondent. Respondents were duly informed on how the study will be conducted by NGOs working in partnership MSM. Participants were informed that blood swab testing for HIV will be conducted. Study staff ensured that the anonymity of participants in this sero-epidemiological survey was respected. All surveillance data was kept in a safe place. Each participant was given an identification number. In order to safeguard anonymity, all the blood samples, data collection tools and administered questionnaires were identified just with a code and an identifier of the center. This made it possible to match the interview questionnaire with the results of the laboratory tests. The specimens were destroyed immediately after the study. There was no register bearing names or other personal details. All databases were protected with strong passwords.

**Data entry and analysis**: Data was inputted into an Epidata database and exported to STATA^®^ 9.0 for analysis. The results were presented in proportions with a confidence interval of 95%. Qualitative variables were compared using the chi-2 or Fisher's exact tests and the mean or median was compared using Student's t-test or variance analysis or the Kruskall Wallis nonparametric or Wilcoxon tests. Univariate and or multivariate logistic regression analyses were also conducted in order to study the relationship between the dependent variable (HIV infection) and the explanatory variables (age, education level, and risky behavior) so as to study the determinants of HIV infection. In this model, the variable, center or region, was systematically incorporated as an imposed variable.

## Results

**Socio demographic characteristics**: In total, 496 MSM were involved in this study, with 43.35% in Lome, the capital of Togo. The average age of MSM interviewed was 23 and an interquartile range (IQR) of 21-27 years. The study population was mainly made up of Togolese, 97.4%; with at least a secondary school level of education; 89.7%; mostly Christians; 69.2%, and pupils or students; 47.6% (Table 1). The overwhelming majority of MSM; 66.7%, was single and resided alone; living with their families; 25.3%, or lived together a partner; 4.8%. Only 14.7% of MSM said they were engaged in a marital relationship, among which 12.7% were married to men and 2.0% to women. Almost half of the respondents, i.e. 44.6% were members of an MSM association and 34.3% had disclosed their sexual orientation to their relatives.

**Table 1:** Socio-demographic characteristic of MSM

Characteristics	Total (N=496)	Lomé (N=215)	Other Cities (N=281)
**Age (years)**			
Median, IQR	23 [21-27]	22 [21-26]	23 [21-28]
Below 23 years	245 (49.4)	119 (55.3)	126 (44.8)
**Nationality, n (%)**			
Togolese	483 (97.4)	202 (94.0)	281 (100.0)
Other Nationalities	13 (2.6)	13 (6.0)	0(0.0)
**Education Level, n (%)**			
Illiterate	15 (3.0)	6 (2.8)	9 (3.2)
Primary	36 (7.3)	14 (6.5)	2 (7.8)
Secondary	271 (54.6)	132 (61.4)	139 (49.5)
Higher education	174 (35.1)	63 (29.3)	111 (39.5)
**Religion, n (%)**			
Christians	343 (69.2)	168 (78.1)	175 (62.)
Animists	50 (10.0)	15 (7.0)	35 (12.5)
Unbelievers	35 (7.1)	7 (3.3)	28 (10.0)
Moslems	63 (12.7)	20 (9.3)	43 (15.2)
Not specified	5 (1.0)	5 (2.3)	0 (0.0)
**Professional Activities n (%)**			
Pupils/Students	236 (47.6)	105 (48.8)	131 (46.6)
Designer/Artists/ Tailors/Electricians	34 (6.9)	20 (9.3)	14 (5.0)
Other Professions^+^	68 (13.7)	32 (14.9)	36 (12.8)
Jobless	69 (13.9)	25 (1.6)	44 (15.7)
**Matrimonial Status, n (%)**			
Married/ Concubine	32 (6.5)	13 (6.0)	19 (6.8)
Singles	452 (91.1)	198 (92.1)	254 (90.4)
Divorcee and widower	7 (1.4)	3 (1.4)	4 (1.4)
Not specified	5 (1.0)	1 (0.5)	4 (1.4)
**Couple, n(%)**			
No	413 (83.3)	171 (79.5)	242 (86.1)
Yes, with a man	63 (12.7)	32 (14.9)	31 (11.0)
Yes, with a woman	10 (2.0)	4 (1.9)	6 (2.1)
Not specified	10 (2.0)	8 (3.7)	2 (0.8)
**Living with family n (%)**			
Alone	331 (66.7)	141 (65.6)	190 (676)
In couple with spouse	24 (4.8)	12 (5.6)	12 (4.3)
In family	125 (25.3)	58 (27.0)	67 (23.8)
Others	14 (2.8)	4 (1.8)	10 (3.6)
Not specified	2 (0.4)	0 (0.0)	2 (0.7)

+ Merchant / Hairdresser / driver / cook

**Risky behavior**: Of all MSM interviewed, 55.4% took alcohol, while about 30% took alcohol at least three times per week. Also, 11.7% and 1% of MSM respectively consumed cannabis and other forms of intravenous drugs. During the last 12 months, 88.9% of MSM had sexual intercourse with men, especially with Togolese (80%). Also, 24.1% of MSM had sex mainly with Togolese women; 57.7% during the last 12 months. Among the sexual partners of MSM, 80% were at least 35 years old. However, 1.4% of MSM had sex with minors. The main sexual practices declared by MSM interviewed were both penetrating and non-penetrating practices. Penetrating sexual intercourse consisted of 194 (68.1%) active anal intercourse and 84 (37.2%) passive anal intercourse during the last 30 days before the study. Non-penetrating intercourse consisted of 194 (87.5%) oral sex and 182 (12.5%) masturbation or oro-anal intercourse. Some 52.9% of MSM had their last sexual intercourse with an occasional partner. These occasional partners were met in 39.7% of cases in friends' homes and in 22.7% of cases in a closed meeting venue. The last sexual intercourse with a man took place in the house of the respondent, 36.9% or in the house of the partner, 35.6% of cases. During the past 30 days before the study, 68.5% MSM regularly used condoms during active anal sexual intercourse, while 71.9% used them during passive anal sexual intercourse. Condoms were scarcely used for non-penetrating sexual practices; between 28% and 36% during oral sex and between 28% to 57% during oro-anal intercourse. About two thirds, that is, 66.3% of MSM interviewed had already done an HIV test. Among them, 83.9% went for post-test counseling during the collection of their test results.

**HIV prevalence**: Of the 491 MSM that accepted to undergo the test, 64 tested HIV positive, making a prevalence rate in 2015 of 13.0%. HIV prevalence was higher in cities located south of Togo, especially in Lome (22.3%), Aneho 24.1%, and Tsevie 10.6%. It was 0.0% in three cities notably Atakpame, Kara and Sokode. A comparison between the urban center of Lome and all the other cities of Togo reveals a statistically significant difference OR = 4.67 (2.57-8.49) with 22.3% in Lome and 5.7% in all the other cities. The HIV prevalence rate increased with age among MSM, going from 9% among those aged 20 years to 23.4% among those aged 30 years and above (p = 0.002). Factors associated to HIV infection among MSM through a univariate analysis were advanced age especially > 30 years OR = 5.3 95%IC (1.85-15.1), residing in Lome OR = 5.56 95%IC (2.90-10.64) and the fact of having been already tested for HIV OR = 2.63 IC95% (1.18-5.87). Through multivariate analysis, three variables were associated with HIV infection among MSM age especially > 30 years OR = 5.05 95%IC (1.77-14.38), HIV testing history OR = 2.63 95%IC (1.24-5.77) and the town of residence of MSM OR =5.93 95%IC (3.13-11.25) (Table 2). The other factors especially nationality, marital status and belonging to an MSM association were not linked to HIV prevalence.

**Table 2:** Factors associated with HIV infection - Multi-variate Analysis (N=491)

	Initial model				Final model		
	n/N	Odds Ratio	IC to95%	P value	Odds Ratio	IC to95%	P value
**Age**							
<20 years	8/89	1			1		
20-25 years	22/222	1.10	[0.44-2.72]	0.836	1.08	[0.43-2.69]	0.856
25-29 years	19/116	2.30	[0.88-6.00]	0.088	2.22	[0.85-5.79]	0.101
>=30 years	15/64	5.30	[1.85-15.1]	0.002	5.05	[1.77-14.38]	0.002
**City of Survey**							
Other Cities	16/276						
Lome	48/215	5.56	[2.90-10.64]	<0.0001	5.93	[3.1311.2]	<0.0001
**Travelling**							
Never travelled	17/154	1			-	-	-
Has travelled abroad	15/147	0.72	[0.32-1.60]	0.425	-	-	-
Not specified	32/173	1.09	[0.52-2.28]	0.810	-	-	-
**Screening history**							
Has never undergone screening	9/149	1			1		
Screened for HIV	49/317	2.63	[1.18-5.87]	0.018	2.67	[1.24-5.77]	0.012
Not specified	6/25	4.40	[1.35-15.34]	0.014	4.29	[1.32-13.89]	0.015

**Ethics approval and consent to participate**: The study protocol was submitted and had the approval by bioethics committee of the Health Minister of Togo. A written and verbal informed consent was obtained of respondent prior to the survey that started. Respondents were duly informed on how the study will be conducted by NGOs working in partnership MSM. Participants were informed that blood swab testing for HIV will be conducted.

## Discussion

Our survey allowed us to assess the behaviors of MSM and measure the prevalence of HIV among them in Togo. The results of this survey show a decrease of HIV prevalence after 4 years of intervention among MSM (Table 3). The average age of MSM involved in our study was 23 years and the majority of them, 97.4%, were Togolese; this is close to the average age of 24 of MSM, with a proportion of 90.3% among Togolese in 2011 in Togo [10]. In Cameroun, the average age of MSM was also 24 [12] while in Tanzania, the average age of MSM was 23 [13]. The MSM population in Togo is, therefore, a young adult population. Only 14.7% of MSM in Togo were living as couples; 12.7% with a man. This can be explained by the difficulties MSM face in fully displaying their sexual orientations because of stigma linked to homophobia. In Malawi, only 18.1% of MSM interviewed in 2013 had already revealed the sexual practices or orientations to their family members and 31% had had female partners during the past 12 months [14]. The low propensity to reveal sexual practices to relatives is also a possible indicator of the strong stigma associated with homosexuality and HIV infection. From the MSM surveyed, 52.9% had sexual intercourse with casual partners. This shows the risky behaviors that the MSM population expose themselves to in Togo. In fact, MSM are difficult to reach and this could jeopardize the effectiveness of sensitization and prevention actions designed for them. In 2007 in Jamaica, 54.2% of MSM studied had had sexual intercourse with a new partner in the course of the last year and 27.4% declared having had two or three male partners during the past four weeks preceding the study [15]. The consistent use of condoms (often and systematically) depended on the form of sexual practice. In our study, 68.5% of MSM had regularly used condoms during active anal sexual intercourses while 71.9% stated having used them during passive anal sexual intercourses. In Jamaica, 61.8% of MSM reported having used condoms during their last sexual intercourse with two male and female partners and during anal sexual intercourse [15]. Stigmatization of MSM is likely to mitigate access to information on the prevention of HIV/STI and subsequently engender risky behaviors such as the refusal to systematically use condoms during sexual intercourse. However, 66.3% of MSM had already done an HIV test in the past 12 months. This shows that MSM in Togo are more aware of their HIV status. In Malawi, 44.3% of the MSM had never done an HIV test [14].

**Table 3:** Evolution of the main behavioral indicators and HIV prevalence among MSM in Togo between 2011 and 2015

Indicators	2011	2015
1. consistent use of condom during sexual intercourse		
active anal sexual intercourse	21.6%	68.5%
passive anal sexual intercourse	18.1%	71.9%
2. MSM proportion who know their HIV status	59.8%	66.3%
3. HIV prevalence	19.6%	13%

The HIV prevalence rate among MSM involved in our study was 13%. In 2013 in Tanzania, the HIV prevalence rate among MSM in two major cities was 30.2% while in [13] Malawi, the prevalence rate among MSM was 15.4% in 2013 [14]. In Togo, the previous national HIV prevalence study among MSM that was conducted in 2011 revealed a prevalence rate of 19.6% [10]. This study, therefore, shows a downward trend in HIV prevalence among MSM in Togo. The expansion of condom use among MSM between 2011 and 2015 (Table 3) may also explain this lowering of HIV prevalence. Despite this drop, HIV prevalence remains higher among MSM than in the general population that has an HIV prevalence rate of 2.5%. The drop in the HIV prevalence rate among MSM could be partially explained by the general decline in HIV infection in Togo, but most importantly, it could be due to the implementation of specific interventions for this population in Togo during the past 6 years [9]. Bisexual practices noticed among 24.1% of MSM involved in our study could constitute an HIV transmission risk factor to the general population. In fact, since the HIV prevalence rate among MSM is higher than in the general population because of the low rate of condom use, MSM could serve as a bridge for the transmission of HIV to the general population. Advanced age, HIV screening history and the city of residence were connected to HIV prevalence among MSM in our study. HIV screening history and residence in the city of Lome were also linked to HIV prevalence among MSM in 2011 in Togo [10]. In Tanzania, a high HIV prevalence rate among MSM was equally noticed among MSM in the biggest city of the country, Dar es Salaam [13]. In Malawi, the fact of being more than 25 years old was associated with HIV prevalence among MSM [14] and in Kenya in 2007, advanced age was also associated with HIV prevalence among MSM [16]. More efficient means need to be sought to decrease the HIV prevalence rate that is currently high among MSM in Togo. Measures to reduce their social vulnerability, fight stigma and discrimination also need to be designed in order to allow them live their sexuality without risks.

## Conclusion

This survey confirms the high HIV prevalence rate among MSM in Togo, with a prevalence rate that is 5 times higher than the national. It also shows a decreasing trend in new infections in this group since (19.6% in 2011 vs 13.1% in 2015) after strengthening the implementation of a prevention and care program. But these results should be consolidated by effectively fighting against stigma and discrimination that limit access to prevention and care services for these HIV-vulnerable populations.

### What is known about this topic

- HSH are vulnerable populations for HIV;
- HIV prevalence is higher in MSM than general population;
- MSM are hard-to-reach and marginalized population.

### What this study adds

- Second study of the kind in Togo concerning this key population of MSM;
- Knowledge of HIV prevalence in HSH population;
- Identify strategies for reducing HIV prevalence in this population.

## Competing interests

The authors declare no competing interests.
